# Influence of the Micro-Nanostructuring of Titanium Dioxide Films on the Photocatalytic Degradation of Formic Acid under UV Illumination

**DOI:** 10.3390/nano12061008

**Published:** 2022-03-18

**Authors:** Nicolas Crespo-Monteiro, Marwa Hamandi, Maria Alejandra Usuga Higuita, Chantal Guillard, Frederic Dappozze, Damien Jamon, Francis Vocanson, Yves Jourlin

**Affiliations:** 1 Laboratoire Hubert Curien, Université de Lyon, UMR CNRS 5516, 42000 Saint-Etienne, France; maria.usuga@univ-st-etienne.fr (M.A.U.H.); damien.jamon@univ-st-etienne.fr (D.J.); francis.vocanson@univ-st-etienne.fr (F.V.); yves.jourlin@univ-st-etienne.fr (Y.J.); 2 University Lyon, University Claude Bernard, CNRS, IRCELYON, UMR5256, 69626 Villeurbanne, France; marwa.hamandi@ircelyon.univ-lyon1.fr (M.H.); chantal.guillard@ircelyon.univ-lyon1.fr (C.G.); frederic.dappozze@ircelyon.univ-lyon1.fr (F.D.)

**Keywords:** sol-gel, micro-nanostructuring, formic acid, photocatalysis

## Abstract

Surface micro-nanostructuring can provide new functionalities and properties to coatings. For example, it can improve the absorption efficiency, hydrophobicity and/or tribology properties. In this context, we studied the influence of micro-nanostructuring on the photocatalytic efficiency of sol-gel TiO_2_ coatings during formic acid degradation under UV illumination. The micro-nanostructuring was performed using the UV illumination of microspheres deposited on a photopatternable sol-gel layer, leading to a hexagonal arrangement of micropillars after development. The structures and coatings were characterized using Raman spectroscopy, ellipsometry, atomic force microscopy and scanning electron microscopy. When the sol-gel TiO_2_ films were unstructured and untreated at 500 °C, their effect on formic acid’s degradation under UV light was negligible. However, when the films were annealed at 500 °C, they crystallized in the anatase phase and affected the degradation of formic acid under UV light, also depending on the thickness of the layer. Finally, we demonstrated that surface micro-nanostructuring in the form of nanopillars can significantly increase the photocatalytic efficiency of a coating during the degradation of formic acid under UV light.

## 1. Introduction

The development and improvement of surface coatings is a major challenge in industry today because materials are used in many strategic sectors, including health, transport, housing, energy, and the production of luxury goods [1,2,3,4]. Metal oxides are an important class of materials that can meet this need thanks to their chemical, mechanical, and thermal stability, and thanks to their physical and chemical properties (e.g., SiO_2_, ZnO, TiO_2_) [5,6,7,8,9,10]. Titanium dioxide (TiO_2_) is the best known and most widely used of these oxides to date. Its main advantages are its optical properties (a high refractive index, and transparency in the visible and NIR range) and its mechanical, physical and chemical properties (photocatalytic properties). The advantages of depositing TiO_2_ coatings in the form of thin films have been demonstrated in many fields, including energy [11], photocatalysis [12,13,14,15,16,17], electronics [18,19], health [20], and optics [21,22,23]. Additionally, the coatings can be micro-nanostructured, thereby giving them new functionalities and properties, e.g., improved absorption efficiency [24], hydrophobicity and enhanced tribological properties [25].

Many studies have consequently been devoted to the development of new techniques to transform flat surfaces into two- or three-dimensional surfaces at the micro-nanometric scale. These techniques are based on two types of approach: bottom-up and top-down. In the bottom-up approach, micro-nanostructures are constructed by additive building processes controlled by the laws of chemistry and physical chemistry [26,27]. These allow the creation of micro-nanostructures with few constraints on scale or resolution, but these approaches are generally limited to simple designs and small surface areas. In the top-down approach, nanostructures are constructed from bulk materials that are selectively patterned to remove some of the bulk, as in optical lithography [21,28,29]. The great majority of industrial nanofabrication techniques fall into this category.

Among the top-down approaches, microsphere photolithography or colloidal lithography (CL) is an inexpensive and promising way to produce regular and homogeneous arrays of micro-nanostructures of different sizes on a planar or cylindrical substrate [29,30,31]. This technique uses microspheres deposited on the surface of the films (usually using a Langmuir–Blodgett method) as micro-lenses to create photonic nanojets under the microspheres. Depending on the material involved, the photonic nanojets then create nano-pillars or nano-holes under the microspheres. The periodic microstructure is thus determined by the quasi hexagonal arrangement of the monolayer of the microspheres deposited on the layer. As we showed in previous studies, this technique can be applied directly to micro-nanostructured titanium oxide coatings [28,29,30].

In the present study, we used colloidal lithography to investigate the influence of patterning on the photocatalytic efficiency of sol-gel TiO_2_ films. The sol-gel approach is a so-called “soft chemistry” technology, which allows the realization of glassy material such as TiO_2_ without having to resort to high temperatures for its processing [21,29,32]. The material is directly fabricated from a viscous solution that can be easily deposited (e.g., by dip-coating or spin-coating) on small or large planar or non-planar substrates [21,29,32], which makes this method very adjustable. Moreover, the sol-gel approach is interesting because the coatings can be photosensitive to UV radiation, which allows direct structuring without having to resort to etching steps [21,29,32], which is one of its main interests. Micro-nanostructured and unstructured coatings were characterized using Raman spectroscopy, ellipsometry, atomic force microscopy (AFM) and scanning electron microscopy (SEM). Their photocatalytic efficiency was evaluated based on the degradation of formic acid under UV illumination. We demonstrated that surface micro-nanostructuring in the form of nanopillars can significantly increase the photocatalytic efficiency of a coating during the degradation of formic acid under UV light due to both light absorption and the specific area thanks to a direct micro-nanostructuring sol-gel layer with colloïdal lithography.

## 2. Materials and Methods

### 2.1. Chemicals

The thin films used in this study were manufactured from a sol-gel at room temperature using the procedure previously described in [21,32]. Briefly, the sol-gel was composed of a mixture of two sols with different reactivities. The first sol was prepared by reacting titanium tetra iso-propoxide (TIPT from Sigma-Adrich, St. Quentin Fallavier, France), deionized water, hydrochloric acid (HCl from Carl Roth, Lauterbourg, France) and butanol (BuOH from Acros Organics, Fischer scientific, Illkirch-Graffenstaden, France) with a TIPT/H_2_O/HCl/BuOH molar composition of 1/0.13/0.82/23.9. The second sol was prepared from TIPT, methanol (MeOH from Sigma-Aldrich, St. Louis, MO, USA) and benzoylacetone (BzAc from Sigma-Aldrich) with a TIPT/MeOH/BzAc molar composition of 1/0.75/20.4 moles. The TiO_2_ sol-gel was obtained by mixing the two sols with a TIPT-BzAc at a molar ratio of 0.6.

### 2.2. Elaboration of the Photocatalyst Thin Films

#### 2.2.1. Unstructured TiO_2_ Films 

The sol-gel layers were deposited on a flat silica substrate (with a diameter of 2 inches, NEYCO Vacuum & materials, Vanves, France) by spin-coating at 3000 rpm, and were then heated in an oven at 500 °C for 3 h. In order to investigate the influence of the thickness of the layer in the photocatalysis study, the process was repeated several times, resulting in multilayers of different thicknesses.

#### 2.2.2. Micro-Nanostructured TiO_2_ Films

**Scheme 1:**
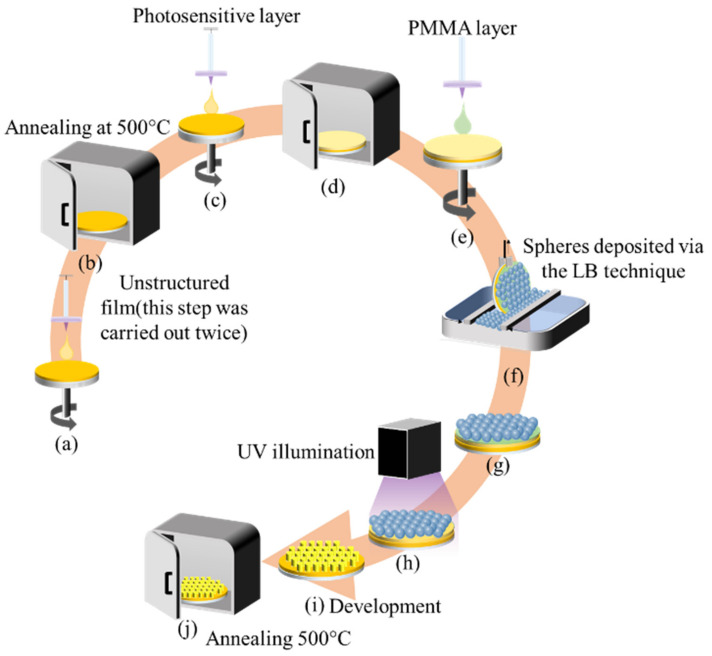
Schematic diagram of the micro-nanostructuring process.

The complex TIPT-BzAc used in the sol-gel solution renders the solution stable and determines its photosensitive behavior under UV light. Under UVA, the complex is partially degraded into insoluble species such as carbonates and/or carboxylates. The non-illuminated areas can then be removed by washing in ethanol and water, while the illuminated zones are insoluble in ethanol and are thus stable. As we already reported in previous studies, this sol can be combined with colloidal photolithography to produce nanopillars [29]. In the present study, we reproduced and used this method on micro-nanostructured TiO_2_ films. A schematic diagram of the micro-nanostructuring process is reported in the Scheme 1.

The sol-gel film was deposited by spin coating at 3000 rpm for 1 min, and was heated at 110 °C for 90 min. This thermal treatment was performed to obtain an amorphous photosensitive xerogel film. In order to preserve the photo-patternable xerogel film of water, a protective layer made of polymethyl methacrylate (PMMA) was deposited on top of the film by spin coating at 5000 rpm for 1 min [29]. Next, a monolayer of silica microspheres (diameter 1 μm) suspended in ethanol (96% *v*/*v*) (Micromod) was deposited using the Langmuir–Blodgett (LB) technique (KSV NIMA LB from Biolin Scientific). The monolayer was deposited with a barrier speed of 2 mm min^−1^, a surface pressure of 35 mN·m^−1^, and a withdrawal speed of 1 mm·min^−1^. The sample was then exposed to homogenous UV illumination at a wavelength of 365 nm for 10 min. Each microsphere of the monolayer behaves like micro-lens by focusing UV light, creating a photonic nanojet in form of concentrated light underneath the microspheres, the size (length and width) of which depends on the spheres’ diameter and exposure wavelength. These nanojets degrade the TIPT-BzAc complex and render the illuminated areas insoluble in ethanol [29]. After the exposure of the microspheres to the UV light, the sample was processed using a three-step procedure based on successive washings in chloroform, ethanol, and deionized water, for 1 min each. Chloroform dissolves PMMA and the untreated xerogel layer, ethanol washes the sample, and water stabilizes the illuminated areas. The entire protocol produced periodic nanopillars arranged in a 2D, hexagonal, periodic pattern [29]. Finally, the sample with the xerogel nanopillars was heated in an oven at 500 °C for 3 h in order to obtain TiO_2_ anatase nanopillars.

### 2.3. Characterization

Both films were characterized before and after annealing at 500 °C. Spectroscopic ellipsometry measurements (UVISEL from Horiba Jobin Yvon Kyoto, Japan, associated with DeltaPsi2 software Jobin Yvon) were taken in order to determine the thickness, refractive index (n) and extinction coefficient (k) of the TiO_2_ thin films. The ellipsometry measurements were performed at an angle of incidence of 60°, in the 0.99 to 4.4 eV spectral range, with a 0.02 eV step. The optical model consists of two layers on a semi-infinite glass substrate. The first layer is made of the material under study (TiO_2_). The second layer is considered to be a roughness layer, with a 50–50% mixed dielectric function between the void and the dielectric function of the first layer. We used the double Tauc Lorentz dispersion formula described in the reference [33] because we had interband aborption in the TiO_2_ thin film. In order to complete the characterization of the film, the thicknesses were also measured with a profilometer (Bruker Dektak XT from Bruker company, Billerica, MA, USA).

Atomic force microscopy (AFM) (Dimension Icon from Bruker) and scanning electron microscopy (NovananoSEM200 FEI with a HELIX detector) were used to characterize the profile of the micro-nanostructured films. The amount of TiO_2_ in the coatings was determined by inductively coupled plasma- optical emission spectrometry (ICP-OES) using an Activa (Jobin Yvon) instrument. The samples were dissolved by acid attack (H_2_SO_4_ (1 mL) + HNO_3_ (4 mL) + HF (0.5 mL) in 25 mL water) and heated at 150 °C for 12 h.

### 2.4. Photocatalytic Experiments 

**Figure 1:**
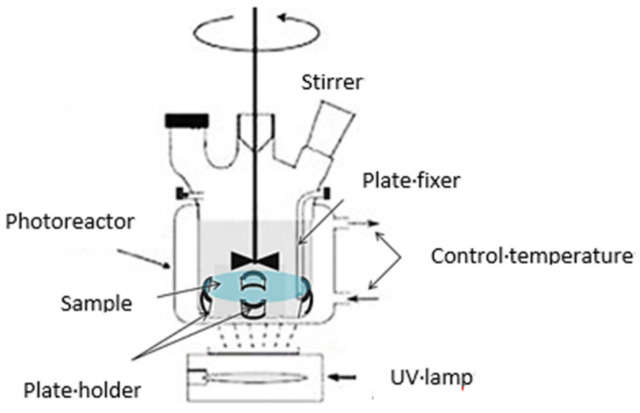
Sketch of the set-up used for the photocatalytic tests.

The supported titania samples were placed on a four quarter-moon shaped glass holder about 1 cm from the bottom of a cylindrical Pyrex photoreactor equipped with a bottom optical window (diameter ca. 5.5 cm) which was open to the air (Figure 1). A radiation flux of 6 mW.cm^−2^ provided by an 18 W PLL lamp with a maximum wavelength of 365 nm was used for all of the photocatalytic tests. The radiation flux was measured with a VLX-3W radiometer and a CX-365 detector (UV-A).

The light absorbed by the TiO_2_ deposit was assessed by measuring the irradiance transmitted through the glass support impregnated with different TiO_2_, coatings, and then subtracting the irradiance transmitted through the support alone.

Aliquots (120 mL) of an aqueous solution containing 50 ppm (1086 μmol/L) formic acid (FA), used as a model pollutant, were continuously stirred on a mechanical stirrer (Mixel TT, 500 tr min^−1^, from Mixel, Dardilly, France). Before irradiation, the stirred solution was kept in the dark for 30 min to reach the adsorption equilibrium, after which the solution was irradiated, and 0.5 mL samples were collected at regular intervals and analyzed by HPLC. 

The formic acid HPLC analyses were performed using a VARIAN system (Agilent Technologies, Les Ulis, France) equipped with a UV-Vis detector and a 300 mm × 7.8 mm carbohydrate analysis column (COREGEL-87H3 from Interchim, Montluçon, France). The mobile phase was a H_2_SO_4_ solution (5 × 10^−3^ mol L^−1^) and the flow rate was set at 0.7 mL min^−1^. The detection wavelength was set at 210 nm.

## 3. Results and Discussion

### 3.1. Characterization of the Coated Surfaces

**Figure 2:**
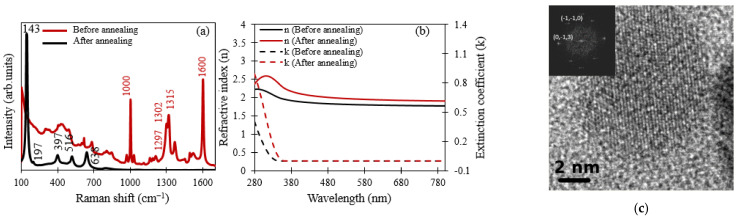
(**a**) Raman spectra. (**b**) Refractive index and extinction coefficient of the film before and after annealing at 500 °C. (**c**) HRTEM micrograph of an anatase TiO_2_ nanocrystallite (inset: the corresponding FFT showing the (−1, −1, 0) and (0, −1, 3) directions of the anatase TiO_2_ tetragonal structure).

Before annealing at 500 °C, the unstructured film was homogenous and transparent in the visible range. Based on profilometry and ellipsometry, the film was estimated to be to be 300 nm thick. The Raman spectrum (Figure 2a) showed multiple peaks but none for crystallized TiO_2_. Following Oda et al. [34], the peaks at 1600 and 1000 cm^−1^ were attributed to the 8b and 12 vibration modes of the phenyl group of BzAc, and the peaks at 1297 and 1315 cm^−1^ were attributed to the symmetric vibrations of the C=C=C bond of the BzAc molecules of the TIPT-BzAc chelated ring. The refractive index (n) of the layer before annealing (Figure 2b) was 1.8 in the visible–NIR range, and the extinction coefficient (k) was close to 0 in this wavelength range, confirming low light absorption (Figure 2b), but below 350 nm the extinction coefficient (k) increased to reach 0.44 at 280 nm. This behavior indicates the absorption of the incident wavelength of the film, which is all the higher as the wavelength decreases below 350 nm. After annealing at 500 °C, four dominant peaks were indeed observed at 142, 396, 516 and 637 cm^−1^ on the Raman spectrum that did not exist before annealing. These peaks were assigned to Raman active modes of anatase TiO_2_ (Figure 2a), as illustrated in the references [35,36]. A small Raman signal was also detected near 800–850 nm and below 400 nm, which could mean that some organic compounds are always present. High-resolution transmission electron microscopy (HRTEM—Jeol 2010F TEM, equipped with a field-emission gun from JEOL Europe SAS, Croissy sur Seine, France) also confirmed the presence of anatase TiO_2_ nanocrystallites in the film, as illustrated in Figure 2c.

The profilometer and the ellipsometry measurement indicated the densification of the film, the thickness of which decreased to around 50 nm. The phase change of the film and its densification led to an increase in the refractive index to around 2.1 in the visible–NIR range, and the extinction coefficient remained nearly zero in this range (Figure 2b). However, the film began to absorb at 360 nm, and reached an extinction coefficient of 0.95 at 280 nm, highlighting the better absorption of the films at these wavelengths after annealing. For the present study, the thickness of the unstructured film was adjusted by making multilayer deposits. 

### 3.2. Photocatalytic Performance for Formic Acid Degradation

#### 3.2.1. Effect of Annealing

Table 1 shows the photocatalytic degradation of FA in the presence of TiO_2_ films prepared by annealing at 500 °C and without annealing. In the non-annealed TiO_2_ film, the disappearance rate of the formic acid was negligible, in agreement with the Raman analysis showing the presence of peaks characteristic of organic compounds, especially BzAc, and no anatase phase. However, the small disappearance rate observed in the absence of annealing suggests that a very small proportion of TiO_2_ anatase existed but was not detected by Raman analysis.

After annealing at 500 °C, TiO_2_ crystallized well to the anatase phase with good activity, fulfilling the requirement for a well-crystalized TiO_2_ phase. Actually, the crystallization of TiO_2_ to the anatase phase is one of the more important parameters in photocatalysis [37,38]. 

#### 3.2.2. Effect of the Film Thickness and Light Absorption on the Photocatalytic Activity

**Figure 3:**
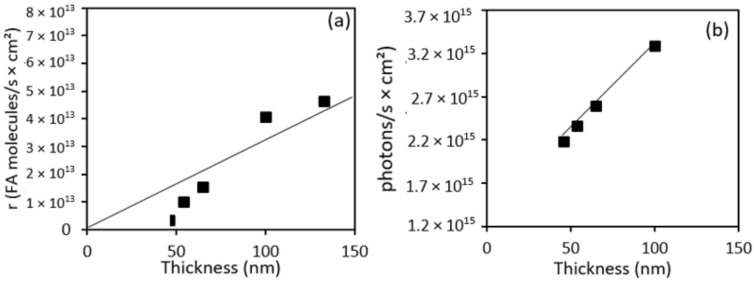
(**a**) FA disappearance rate as a function of the thickness of the unstructured TiO_2_ film. (**b**) Photons absorbed as a function of the TiO_2_ thickness.

Increasing the thickness of the TiO_2_ film increased the degradation rate of FA (Figure 3), as was already reported in the literature in the case of other pollutants [38,39,40]. This correlation is explained by the absorption of light that increases linearly to the TiO_2_ thickness in this range of thickness (Figure 3b). Actually, by increasing the absorption light, more (e−, h+) are generated, thereby favoring the degradation of formic acid. From this result, the quantum yield can be estimated as the ratio of the photocatalytic degradation rate to the absorbed light.

**Figure 4:**
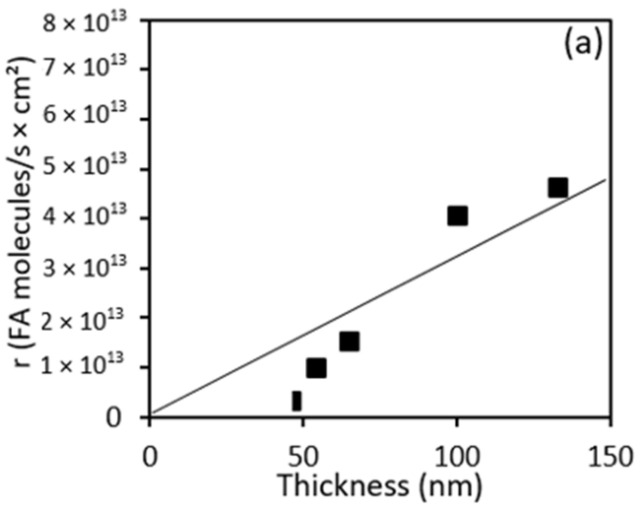
Disappearance rate of FA as a function of the photons absorbed.

By drawing the disappearance rate as a function of light absorption (Figure 4), a quantum yield of 3.3% was obtained, considering the slope of the curve. However, it is important to note that the number of absorbed photons is difficult to estimate experimentally, owing to reflection; as such, the value is probably underestimated.

#### 3.2.3. Effect of Micro-Nanostructuring on the Films

**Figure 5:**
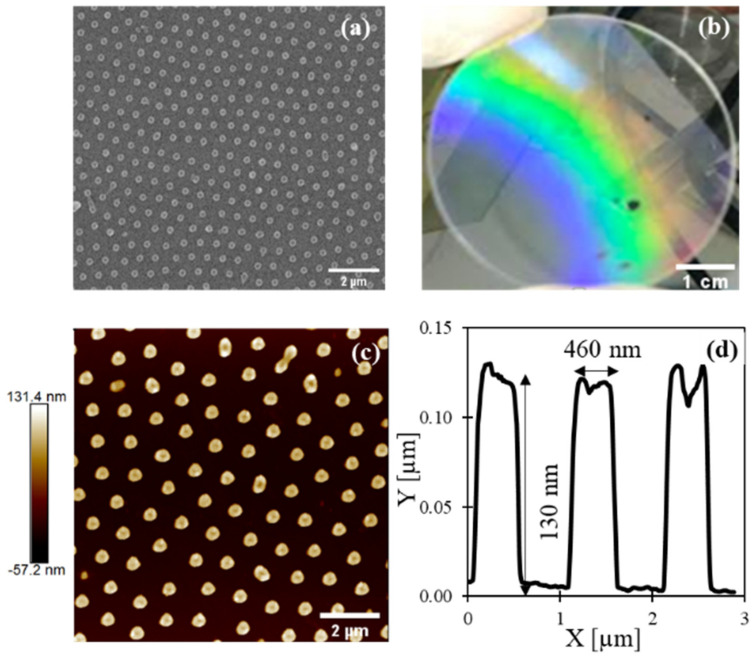
(**a**) SEM top view image. (**b**) Optical photograph of the micro- nanostructured sample. (**c**) AFM images. (**d**) Profile of the nanopillars.

For micro-nanostructuring, the colloidal photolithography of the thin films was performed on an unstructured anatase TiO_2_ film with a thickness of 140 nm (Scheme 1). For this purpose, a sol-gel photosensitive film was deposited on top of the unstructured film of TiO_2_ anatase. After the deposition of the silica spheres (diameter 1 μm) using the Langmuir–Blodgett technique, UV light at a 365 nm wavelength illuminated the sample to create photonic nanojets under the silica nanospheres. These nanojets degraded the TIPT-BzAc complex and rendered the illuminated areas insoluble in ethanol. After development and annealing at 500 °C for 3 h, anatase nanopillars were obtained on the surface of the film (Figure 5a,c,d).

The optical photograph (Figure 5b) shows visible light over the entire sample, confirming that the structuring of the whole surface of the sample has indeed occurred. The SEM top view image and AFM profile (Figure 5b,d,e) indicate a homogeneous micro-nanostructure of nano-pillars with diameters of around 460 nm and a height of around 130 nm. The nano-pillars are organized in a hexagonal structure of which the period is 1 μm. A 10 × 10 μm^2^ area contains around 100 nano-pillars. The contact surface between the TiO_2_ and the formic acid is estimated as being equivalent to the surface of the sample (10 μm × 10 μm) to which the sides of the nano-pillars ((2 × π × nano-pillar radius × nano-pillar height) × number of nano-pillars) are added. This calculation showed that, in the micro-nanostructured film, the contact surface increased by 118.8 μm^2^. When we compared this surface with the contact surface generated by an unstructured film of the same thickness, we found that adding an unstructured film of this thickness increased the contact surface by 105.2 μm^2^ (10 μm × 10 μm + 4 × 10 μm × film thickness). For the sake of comparison, we found that the micro-nanostructured film increased the contact area by about 11% compared to an unstructured film.

**Figure 6:**
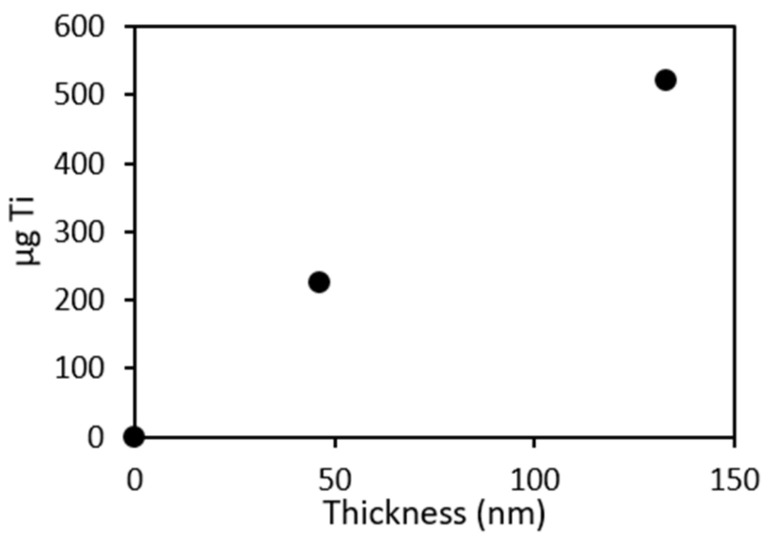
Amount of Ti present in an unstructured TiO_2_ film as a function of its thickness.

As the estimation of the light absorption by micro-nanostructured film is complicated and is impossible to compare to the thickness of film, in order to evaluate the photocatalytic efficiency of the micro-nanostructured film, first we checked for proportionality between the thickness and the amount of TiO_2_. Figure 6 confirms that the amount of Ti increases proportionally with the thickness, as we already found in a previous study [38].

**Figure 7:**
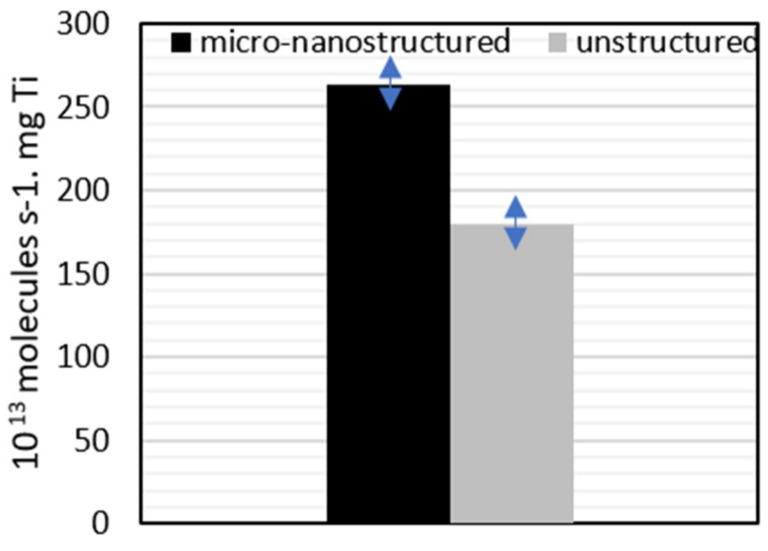
Disappearance rate of FA per μg of Ti obtained for micro-nanostructured and unstructured TiO_2_ films.

Thus, as the degradation rate is proportional to the thickness, the rate can be considered to be proportional to the amount of Ti, and the photocatalytic efficiency of micro-nanostructured and unstructured films can be compared by calculating the disappearance rate of FA per μg of Ti (Table 2, Figure 7). 

We clearly demonstrated that micro-nanostructured film is about 46% more efficient than unstructured film, whereas the amount of Ti only increased by about 10%. However, this improvement may be due to different phenomena, in particular the increase in the contact surface area of Ti with the pollutant, or the improved light absorption. As suggested in another work, the improvement of the light absorption can increase the mean photochemical efficiency [41]. A 20 nm thick anatase layer conformally deposited onto a wavelength-scale two-dimensional periodic photonic lattice allowed the increase of the photochemical efficiency by a factor of up to 5.7 compared to the flat references [41]. 

## 4. Conclusions

In this study, we demonstrated that, compared to flat TiO_2_ films, the surface micro-nanostructuring of a TiO_2_ film significantly improved the photocatalytic efficiency of the films in formic acid degradation under UV light. 

When sol-gel TiO_2_ films are unstructured and not annealed at 500 °C, their effect on the formic acid degradation under the UV light is negligible, whereas when the films are annealed at 500 °C, which leads to crystallization of anatase phase, they increase the degradation of formic acid. Likewise, increasing the thickness of the TiO_2_ film increases the rate of FA degradation. This phenomenon is explained by light absorption, which increases linearly with the thickness of TiO_2_ in this thickness range. Indeed, by increasing the light absorption, more (e−, h+) are generated, thereby enhancing FA degradation. From this result, we estimated the quantum yield to be 3.3%, but due to measurement difficulties, this ratio was probably underestimated. 

Colloidal photolithography was performed to obtain the micro-nanostructuring of an unstructured anatase TiO_2_ film with a thickness of 140 nm. The resulting nano-pillars were homogeneous, and were organized in a hexagonal structure with a 1 μm period. The diameters of the pillars are around 460 nm, and their height is around 130 nm. The photocatalytic efficiencies of micro-nanostructured and unstructured films were compared by calculating the rate of FA disappearance per μg of Ti. The results indicated a photocatalytic efficiency of 180 × 10^13^ molecules/s/mg Ti for the unstructured film and 263 × 10^13^ molecules/s/mg Ti for the micro-nanostructured film. It is clear that the micro-nanostructured film is more efficient than the unstructured film, whereas the amount of Ti increased only slightly. It is worth noting that the 46% improvement in the overall rate of FA can not only be explained by the increase in the surface area available for exchange due to TiO_2_ nanopillars (estimated at roughly 11%) but also by the improved light absorption owing to the micro-nanostructured film in the range of the wavelength. Thus, both phenomena must be addressed.

The further development of the micro-nanostructuring of TiO_2_ anatase thin films requires an increase in both light absorption (photon confinement) and in specific surfaces with an optimized micro-nanostructured pattern. Such developments will depend on rigorous optical modeling and topographically (geometrically) based approaches for the surface.

## Figures and Tables

**Table 1:** Disappearance rate of formic acid obtained in the presence of an unstructured TiO_2_ film before and after annealing at 500 °C.

TiO_2_ Film	r (Molecules/s.cm^2^)
Before annealing	(1.6 ± 0.5) 10^13^
After annealing	(7.4 ± 0.5) 10^13^

**Table 2:** Comparison of micro-nanostructured and unstructured films.

TiO_2_ Film	Ti (μg) for 2 Inches (20.27 cm^2^)	Molecules/s/cm^2^	Molecules/s/mg Ti
Unstructured	521 ± 10	(4.63 ± 0.5) 10^13^	(180 ± 10) 10^13^
Structured	571 ± 10	(7.42 ± 0.5) 10^13^	(263 ± 10) 10^13^
